# *Mycobacterium avium* complex lung infection mimics lung cancer nodules but improves after antibiotic therapy: a case series

**DOI:** 10.3389/fmed.2026.1721002

**Published:** 2026-02-27

**Authors:** Derek Jacobs, John Greene, Lary A. Robinson

**Affiliations:** 1 University of South Florida Morsani College of Medicine, Tampa, FL, United States; 2 Division of Infectious Diseases and Tropical Medicine, Moffitt Cancer Center, Tampa, FL, United States; 3 Division of Thoracic Oncology, Moffitt Cancer Center, Tampa, FL, United States

**Keywords:** case report, lung cancer, lung nodules, macrolide antibiotics, *Mycobacterium avium* complex

## Abstract

**Background:**

Infection with non-tuberculous mycobacterium species commonly can present with lung nodules mimicking lung cancer on routine chest imaging, leading to unnecessary invasive procedures.

**Case series:**

The authors report three representative cases presenting with minimal or no symptoms where an incidental probable *Mycobacterium avium* complex (MAC) infection was discovered, mandating evaluation for a potential malignancy. After suspecting that the nodules were likely infectious, the patients were empirically treated with azithromycin alone or in combination with other antibiotics, resulting in an improved appearance of these nodules on radiographic imaging in size, density, or both, documenting an infectious etiology, thereby preventing more invasive studies and/or surgery.

**Conclusion:**

These cases represent typical examples of an increasingly common infection that can be mistaken for lung cancer, particularly in endemic areas such as the Southeast United States. Infectious etiologies, especially non-tuberculous mycobacteria, are capable of mimicking lung cancer. An empiric, 3-month course of azithromycin for suspected MAC lung infection resulting in significant nodule size reduction verifies that it is infectious and not malignant, thereby preventing invasive, potentially morbid, and expensive evaluations, including unnecessary surgery.

## 1 Introduction

An estimated 1.6 million people every year in the US are found to have a pulmonary nodule (1). Although the rates of malignancy in lung nodules detected by imaging modalities are estimated to be at most 3–5% (2), the detection of lung nodules is frequently followed by further testing to elucidate their diagnosis (1). Considering that lung cancer is the leading cause of cancer death globally (3), the discovery of a potential malignancy is a significant finding requiring prompt evaluation. Infectious agents are a well-documented cause of lung nodules that can mimic lung carcinoma (4) and lead to invasive and unnecessary testing and/or surgery.

Non-tuberculous mycobacteria, such as *Mycobacterium avium* complex (MAC), are one such infectious agent that have previously been shown to mimic lung cancer in a variety of imaging modalities (5). However, documentation of the clinical presentation and resolution of these cases remains lacking. The authors present three representative cases of patients who were referred to our indeterminate lung nodule clinic with suspected lung cancer for surgery, and were discovered to have carcinoma-mimicking lung nodules caused by suspected or proven MAC infections. The nodules substantially improved or resolved after an empiric course of azithromycin or azithromycin combination therapy.

## 2 Illustrative cases

### 2.1 Case 1

**Figure 1:**
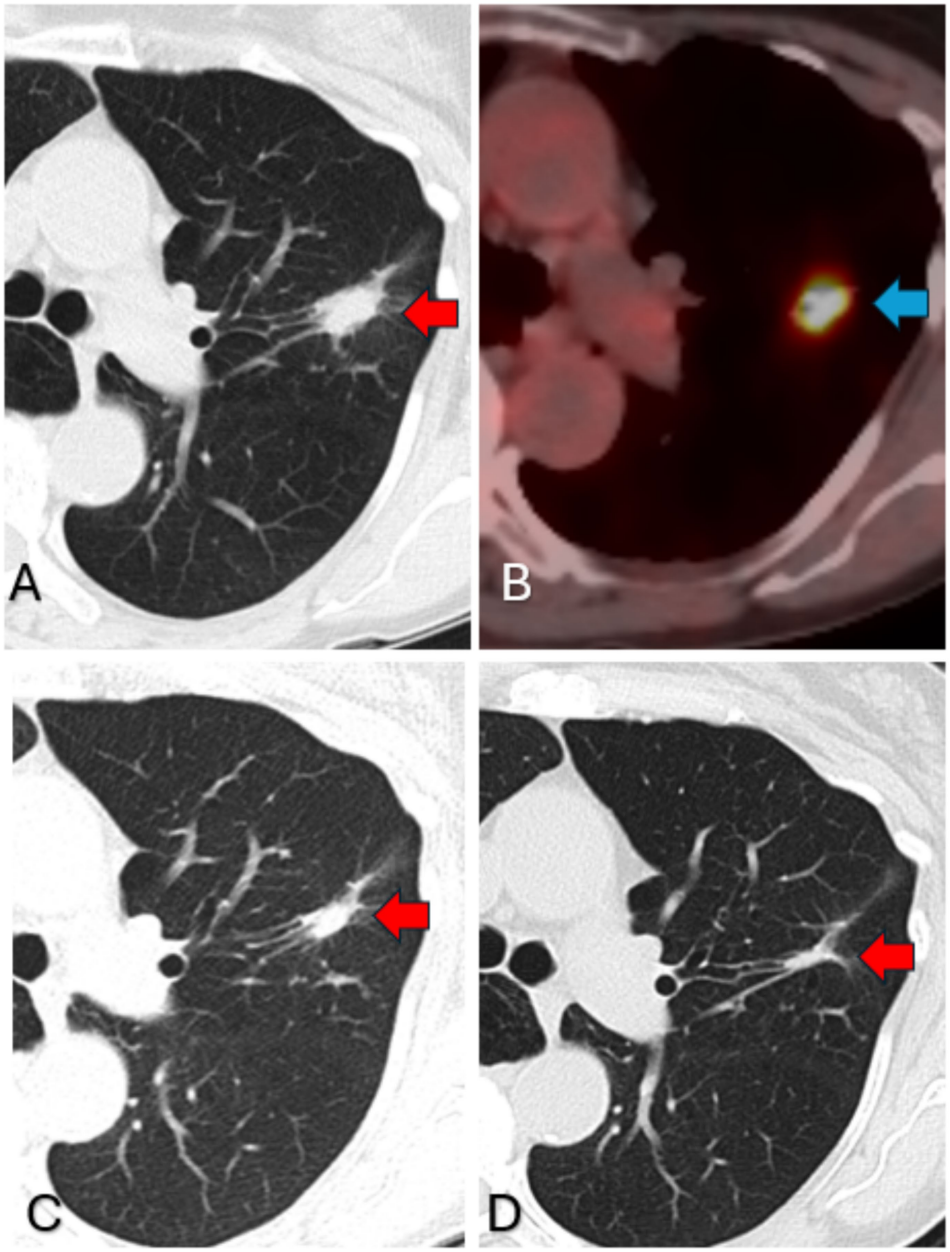
**(A)** Chest CT 18 September 2017 and **(B)** FDG-PET October 2017 prior to treatment. **(C)** 18 October 2018 1 year after treatment and **(D)** 21 November 2024 6 years later.

A 73-year-old Caucasian female former smoker with a history of mild emphysema presented to the indeterminate lung nodule clinic after having an abnormal chest x-ray that revealed waxing and waning lung nodules 5 years previously. Outside a brief, isolated incident of hemoptysis 1 year prior, the patient reported no respiratory symptoms and had an Eastern Cooperative Oncology Group (ECOG) performance status of 0. Her physical examination was entirely normal. She had no significant comorbidities. Additionally, the patient’s social history included a 36-pack-year smoking history, although the patient quit smoking over 15 years ago. Chest computed tomography (CT) and fluorodeoxyglucose positron emission tomographic (FDG-PET) scan at presentation revealed a glucose-avid 1.9-cm left upper lobe nodule (standard uptake value [SUV]: 4.8), as well as a tree-in-bud cluster of nodules in the contralateral medial right middle lobe (Figures 1A,B).

The patient underwent a transbronchoscopic biopsy of the nodule, which revealed granulomatous disease. Bronchoalveolar lavage cultures also revealed the growth of MAC. However, the patient was referred to the thoracic surgery clinic for a possible upper lobectomy due to the concern that there was an underlying lung cancer. The thoracic surgeon fortunately recommended against the procedure and prescribed 250 mg per day of azithromycin along with probiotics. After 3 months of azithromycin, the patient returned for a follow-up, and a repeat chest CT showed a significant decrease in nodule size.

At this time, an infectious disease consultation was requested, and the patient was advised to continue azithromycin monotherapy for an additional 9 months to complete a 1-year course. A repeat chest CT scan after completion of a 1-year azithromycin course showed continued reduction in the size of the upper left lobe nodule with some persistent bronchiectasis and tree-in-bud nodules, in addition to decreased size of the multiple nodules in the medial segment of the right middle lobe at 1 year (Figure 1C). She had no side effects from the azithromycin monotherapy. The patient was quite pleased with the result, especially the fact that she did not need to have a non-therapeutic lobectomy that had been initially recommended. At this time, the azithromycin therapy was discontinued, and the patient was followed with yearly chest CT scans, with the expectation she might develop recurrent similar nodules over the succeeding years, but fortunately 6 years later, this never occurred (Figure 1D).

### 2.2 Case 2

**Figure 2:**
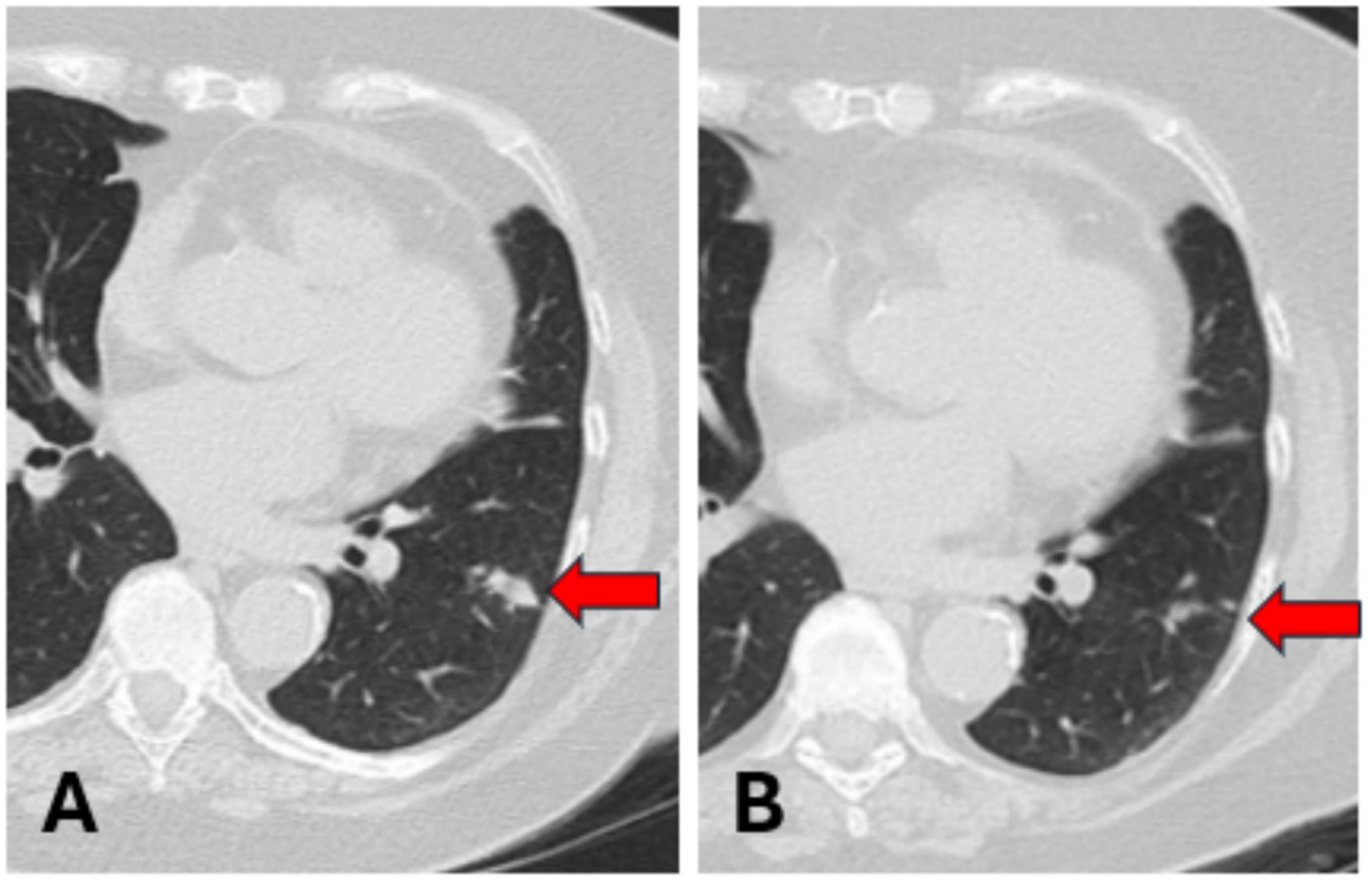
**(A)** Pre-treatment CT scan. **(B)** CT scan after 3 months of azithromycin therapy.

An 81-year-old Caucasian female with a 39-pack-year history of tobacco use and moderate emphysema presented with a 6-month history of mild dyspnea, cough with white sputum, and multiple lung nodules. Her physical examination was normal except for slightly decreased breath sounds bilaterally. Her other comorbidities included hypertension, hyperlipidemia, arthritis, gastroesophageal reflux disease, and moderate obesity (body mass index [BMI] 32). When the productive cough first began, she underwent a chest x-ray, which revealed some nodular densities. She was treated with a 7-day course of levofloxacin, which resolved the majority of the respiratory symptoms. A follow-up chest CT scan revealed that the small nodules in the right middle lobe had decreased in size, but a new left lower lobe density was discovered. The patient was empirically prescribed a 5-day course of azithromycin and followed up after 2 months, when imaging revealed that the left lower lobe spiculated density had increased to approximately 0.3 cm in diameter (Figure 2A).

The patient was then referred to our thoracic surgery indeterminate lung nodule clinic with suspected lung cancer. When the patient was seen upon consultation, her cough had returned, and she was occasionally producing yellow to clear phlegm. Additionally, pulmonary function tests were significantly depressed, with an FEV1 of only 56% of predicted. The patient reported being on several inhaled medications for her emphysema. This patient’s case was discussed at the Thoracic Oncology Conference, and considering the rapid growth of the nodule and the tree-in-bud pattern of the nodules, it was deemed likely that the underlying cause was infectious rather than cancer.

She was prescribed an additional 3-month course of azithromycin 250 mg daily plus a probiotic, which she tolerated well without side effects. Upon completion of the azithromycin, her cough had resolved, and the patient underwent a repeat chest CT scan, which revealed that the left lower lobe nodule had decreased in size by approximately 80% (Figure 2B). The patient had previously declined to have bronchoscopy or needle biopsy for diagnostic purposes. When infectious disease was consulted, imaging findings were consistent with nodular pneumonia with tree-in-bud formations in the left lower and middle right lobes, suggestive of a MAC infection. The remaining nodules appeared stable in size, and the patient was subsequently followed with a chest CT scan 1 year later, which showed no change. The patient was pleased with her treatment outcome, especially because she avoided any invasive procedures.

### 2.3 Case 3

**Figure 3:**
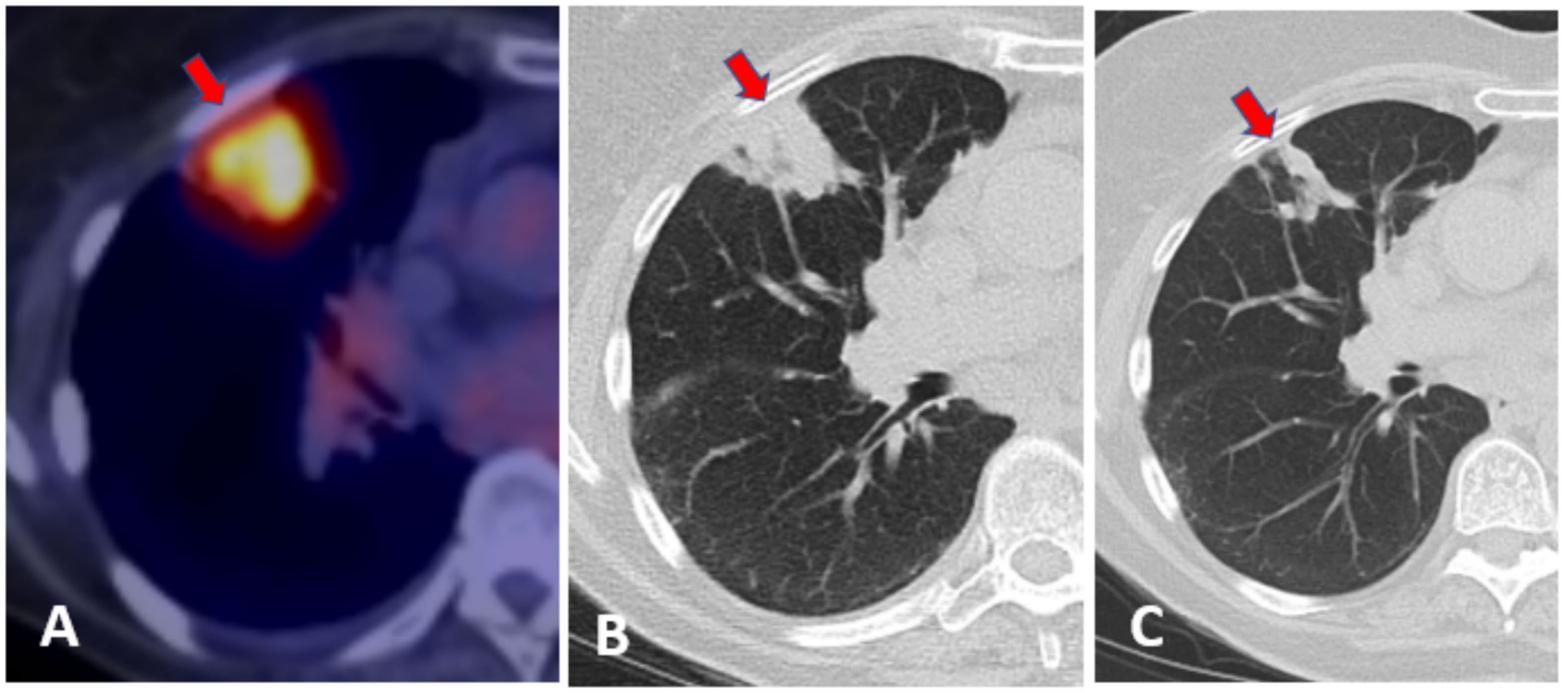
**(A)** Initial FDG-PET scan 27 April 2021. **(B)** Pre-treatment CT scan 9 September 2021. **(C)** Post 6-month treatment CT scan 3 March 2022.

An 85-year-old Caucasian female with significant comorbidities of coronary artery disease, hypertension, obesity (BMI: 35), and emphysema was referred to radiation oncology for a spiculated PET scan-positive (SUV: 5.1) nodule in the right upper lobe of the lung with no significant pulmonary symptoms. Her physical examination was significant for decreased breath sounds and obesity. A core needle biopsy revealed a non-necrotizing granuloma with rare, atypical epithelial cells with negative stains and cultures. Due to concerns that the nodule was actually a small cancer, the patient underwent an empiric stereotactic radiation therapy regimen and subsequently returned every 3 months for CT scans. Upon follow-up, the original nodule had dissolved into an area of hazy fibrosis, but a new, PET scan-positive nodule was discovered in the right middle lobe (Figures 3A,B). A core needle biopsy revealed necrotizing granulomatous inflammation that was negative for acid-fast bacilli, fungi, and other bacteria on staining. However, the culture from the needle biopsy grew MAC that was susceptible to many of the typical antibiotics, including ethambutol and clarithromycin. Additionally, a small left pleural effusion was noted. She was started on 250 mg/day of azithromycin with a follow-up chest CT scan scheduled for 6 months later. After 6 months of azithromycin treatment, the radiologist reported development of the nodule into a more mass-like consolidation and nodular conglomerate in the right middle lobe with improvement of the observed pleural effusion.

At this time, the patient was started on 800 mg/day of ethambutol in addition to the azithromycin, which was continued at the same dosage. After a further 6 months, a chest CT revealed the right middle lobe nodule decreased in size and density and was now visible as clusters of 0.5–1.0 cm nodules (Figure 3C). It was also noted that the pleural effusion had completely resolved. The patient agreed to continue the azithromycin and ethambutol therapy for a further 6 months and then return for reevaluation. Throughout the course of treatment, the patient denied any coughs, fevers, trouble breathing, or other respiratory symptoms, and fortunately, no side effects from the antibiotics. She had no recurrence of symptoms or changes in the cluster of lung nodules on a CT scan 1 year after completing treatment. The patient was pleased with her treatment outcome, particularly because she avoided unnecessary radiation therapy.

## 3 Discussion

**Figure 4:**
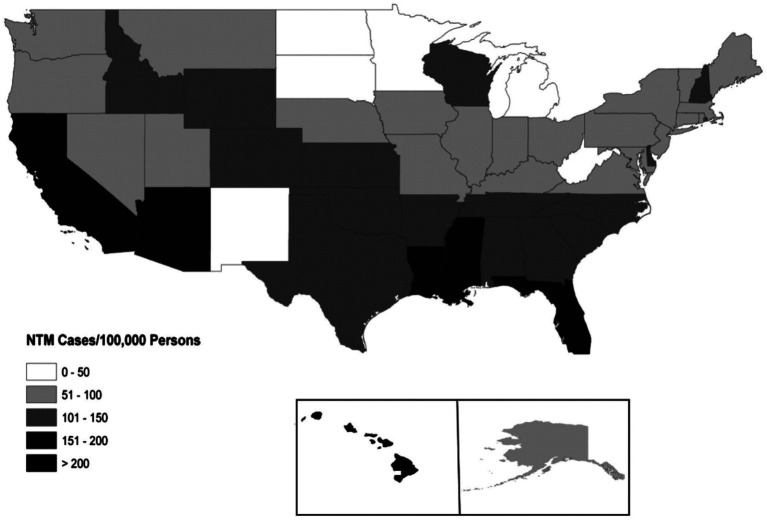
Prevalence of non-tuberculous mycobacterial (NTM) pulmonary infections in Medicare beneficiaries over age 65 years. (Reproduced by permission from the American Thoracic Society, Inc. Am J Respir Crit Care Med, https://www.atsjournals.org/doi/abs/10.1164/rccm.201111-2016OC).

The presence of lung nodules in the lingula segment of the left upper lobe and right middle lobe of the lung caused by MAC in elderly Caucasian females, as illustrated in several of these cases, fits the classical description of Lady Windermere Syndrome (6). This is one of the well-known presentations of MAC characterized by older, post-menopausal women, often never smokers, who tend to suppress their cough due to social norms or a fastidious nature, which allows secretions to accumulate in the airways, creating a favorable environment for infection (6, 7). The older, thin leading lady in Oscar Wilde’s 19th-century play *Lady Windermere’s Fan* was the syndrome’s namesake. Although this infection is uncommon, it appears to be increasing in prevalence in many parts of the world, particularly in warm, humid areas such as Florida, where MAC is endemic (8, 9) (Figure 4, endemic MAC areas).

### 3.1 Pulmonary MAC versus lung cancer

Unfortunately, many patients with lung nodules of probable infectious etiology undergo aggressive follow-up care at similar rates as patients with malignant lung nodules, including procedures such as surgical lung resections, transbronchoscopic biopsy, or transthoracic lung biopsy (10). These procedures carry significant risks, particularly in the usual elderly patients who may be frail and who are often quite reluctant to have an invasive procedure with its risks of pneumothorax, bleeding, and need for a chest tube. CT-guided needle biopsies of lung lesions cause pneumothorax in 26% of procedures and carry a 0.12% mortality rate (11). Navigational bronchoscopy is 79% accurate, similar to transthoracic needle biopsy, but also carries a pneumothorax and bleeding risk that many elderly patients are reluctant to undergo (12).

Nationally, 35% of the lung nodules removed at surgery were benign, with the majority of them found to be an infection (13). Aside from the morbidity and potential mortality of an unnecessary lung resection for an infection, the most recent cost averages $31,900 according to Medicare claims for lung resections (even a “surgical biopsy,” as it is often called), and the majority of patients will have a significant out-of-pocket co-pay (14). Avoiding these procedures is preferable when the nodules have the clinical and radiographic characteristics of an infection rather than malignancy to avoid overtreatment and iatrogenic injury, in addition to the unnecessary medical costs. Nevertheless, routine widespread empiric antibiotic treatment of all nodules is not recommended, although there is a strong trend toward nodule improvement with antibiotics when large clinical series are evaluated objectively (15). Selected antibiotic usage appears to have a benefit in prior lung screening series (16). However, suspected MAC has more specific characteristics that may suggest empiric macrolide therapy may be of significant diagnostic benefit.

However, it is important to recognize that MAC infection can also be present in patients with lung cancer. That is, they are not mutually exclusive. In a study by Tamura et al. (17) of 1,382 untreated lung cancer patients who underwent bronchoscopy, where cultures for mycobacteria were gathered in addition to pathology specimens, 25 (2%) were also positive for MAC or other mycobacteria on culture, most commonly in women smokers of advanced age. The culture-proven MAC and the lung cancer were almost always present in the same lobe, usually with a fibrocavitary or nodule/bronchiectatic disease presentation. They speculated whether the chronic inflammatory response to the MAC infection could lead to lung cancer or whether the presence of the lung cancer allows for the establishment of a MAC in the surrounding bronchioles. It is well described in epidemiological studies that prior pulmonary tuberculosis increases lung cancer risk after many years so that non-tuberculous mycobacteria may also increase that risk (18, 19). Therefore, it is important not to completely dismiss the slight possibility that lung cancer may coexist with the MAC infection, particularly if antimicrobial failure occurs (20).

Studies have shown that only 17% of biopsied lung nodules were malignant, whereas 19% of biopsied lung nodules were definitely infectious in origin (21). Lokhandwala et al. (22) reported on a Surveillance, Epidemiology, and End Results Program (SEER) database study that 43.7% of CT-guided lung biopsies are unnecessary and do not follow the National Comprehensive Cancer Network (NCCN) guidelines. Aside from the potential morbidity to patients, the monetary cost of unnecessary procedures is considerable. It was documented that for Medicare patients between 2011 and 2013, the median cost for an *uncomplicated* needle biopsy was $1,071, while in cases with complications, the median cost increased to $14,824 (22). By 2017, the median cost of a needle biopsy had risen to $4,157, according to the SEER database (22). These costs further emphasize the need to recognize the presentation of infectious lung nodules to reduce the number of risky, costly, and unnecessary procedures.

The tendency of MAC to mimic lung cancer has been described previously in various surgical case series of lung resections that were found on the postoperative lobectomy pathology specimen to be MAC (23–25). A learning point from these publications is that “Positive findings in PET scans do not necessarily indicate malignancy, as similar findings can be observed in inflammatory disorders” (23). Unfortunately, these patients underwent a major surgical resection that likely could have been prevented if MAC had been considered and empirically treated as an initial diagnostic technique. As A. Khanani et al. concluded, their experience should “prompt clinicians to maintain a high index of suspicion for MAC even in cases where the radiographic findings deviate from the norm” (25).

Macrolide antibiotics have known anti-inflammatory effects and are known to be successful in treating inflammatory disorders such as pan-bronchiolitis, asthma, bronchiectasis, and cryptogenic organizing pneumonia (15). However, we have found no prior publications discussing the use of empiric macrolide therapy for suspected MAC infections, especially as a diagnostic approach. In fact, in our indeterminate lung nodule clinic, we are commonly using empiric azithromycin therapy for highly selected, asymptomatic or minimally symptomatic lung nodule patients with suspected MAC as a diagnostic test to see if the characteristic lung lesion(s) decrease in size over a short-term follow-up. In the last 6 months of 2025, we have found 307 new lung nodule patients, and we have used a 3-month course of azithromycin in 15 (5%) patients with suspected MAC, with a favorable response in 7 (significant lung nodule size reduction on chest CT scans), and the rest are pending follow-up chest CT scans to evaluate the results.

Obviously, differentiating infectious from malignant nodules is critically important. One key distinction is the evolution of lung nodules over time. Reviews of chest imaging have reported that lung cancers tend to be “associated with a fairly steady or accelerated growth,” and that growth after 1-year follow-up was a strong predictor of malignancy (26). Lung nodules that shrink spontaneously are highly unlikely to be cancer. Additionally, there is evidence that supports the finding that spontaneously shrinking lung nodules are caused by NTM infections (27). Nodule shrinkage in response to empiric antibiotics in *selected* patients is another way of differentiating malignancy from infections (16).

FDG-PET scans cannot reliably distinguish between a benign infectious nodule and a malignant nodule, as both typically exhibit some glucose avidity. This occurs because leukocytes, which mediate the inflammatory response in infectious nodules, primarily use glucose as their main energy source (28). While lung cancer does not respond to antibiotics, bacterial infections, particularly NTM infections, usually decrease in size with appropriate antibiotic therapy (29).

### 3.2 Who is a candidate for empiric azithromycin monotherapy as a diagnostic tool?

Selective antibiotic treatment of a lung nodule(s) suspected to be infectious with antibiotics represents a safe, non-invasive approach to determine whether an infection is the cause of the abnormal chest CT scan findings. However, a thoracic surgeon or pulmonologist who is very experienced in evaluating lung nodules is the best provider to determine whether an indeterminate lung nodule is more likely an infection, perhaps MAC, and that an empiric antibiotic trial, *usually at least 3 months in length*, is indicated. If the nodule does not respond to empiric antibiotics, then an invasive procedure may be performed. If lung cancer is ultimately documented, then the patient can still have potentially curative treatment, and it is highly unlikely that the peripheral cancer stage or chance for cure has been compromised by a 3-month delay of antibiotic treatment. For this reason, whenever possible, patients with indeterminate lung nodules may significantly benefit by referral to a dedicated lung nodule clinic where they can be evaluated by highly experienced providers, and selective empiric antibiotics may be given *if there is a high index of suspicion that an infection is present, rather than a malignancy*.

Although there is significant overlap, there are some radiologic features that can help differentiate MAC infections from malignant nodules (Table 1). One of the classical features of MAC infections is its location preference for the right middle lobe and lingula of the left upper lobe, although the other lobes also are commonly involved (6). MAC and other non-tuberculous mycobacterial infections also frequently present with the radiographic picture of a “tree-in-bud” nodularity that can be useful in identifying nodules of infectious origin (30). However, spontaneous shrinking of nodules or a decrease in size in response to antibiotics such as azithromycin are further radiologic findings that are highly indicative of MAC infection. An empirical course of azithromycin is not intended as curative for presumed MAC infection, but rather it is done as a *diagnostic* measure to see if there is a significant decrease in the size of the nodule.

**Table 1:** Common characteristics of patients with *Mycobacterium avium* complex (MAC) lung infection.

Characteristics	Findings
Patient factors	
Sex	Postmenopausal female, frequently thin
Age	>65 years old
Cough	Dry cough or with occasional clear to yellow mucus
Activity	Commonly works out in the garden or yard.
Location	Lives in an endemic area, especially humid, warm climate
Immune function	May or may not be immunosuppressed
Smoking history	Higher risk if current or former smoker
Fever or night sweats	Rare fevers but some with night sweats (may be difficult to differentiate from menopausal symptoms)
Other symptoms	Rare hemoptysis, weight loss, dyspnea, or fatigue unless severe disease.
Radiographic features	
CT scan	Speculated peripheral infiltrative nodule(s). May have waxing and waning nodules over years. The most common location is the right middle lobe and the left upper lobe lingula.
FDG-PET scan	Low-to-moderate level uptake of glucose (present in infection and in cancer); minimal or no uptake in lymph nodes
Lymphadenopathy	None or minimal
Lesions in other areas of lungs	Nodular densities, especially if a “tree-in-bud” pattern
Evidence of chronic lung disease	Particularly susceptible to MAC, especially bronchiectasis or COPD

Few MAC lung infection patients will have all of these characteristics.

The standard infectious disease recommendation for MAC treatment is triple-drug therapy (macrolide, ethambutol, and rifampin) for 12 months (31). Macrolide monotherapy is discouraged because of the concern for rapid drug resistance, and this has been reported in a few patients in several case studies, all of which involve patients with upper lobe cavitary disease, nodular disease with bronchiectasis, or cystic fibrosis, where there is a high inoculum of organisms (32, 33). However, another case series of 29 patients without human immunodeficiency virus but with fibrocavitary disease and bilateral disease, who were treated for 6 months with azithromycin monotherapy, resulted in 76% of patients with reduced positive sputum and *no* drug resistance during monotherapy (34). A large multicenter study of 88 AIDS patients with disseminated MAC with symptoms and positive blood cultures treated with azithromycin monotherapy resulted in blood culture sterilization of 54% and drug resistance in only one patient (1.1%) (35). Macrolide resistance with monotherapy is a rare event and generally occurs only in high-inoculum Mycobacterium infections found in large cavities and extensive bronchiectasis. However, in lung nodules with granulomata, there is much less chance for resistance since there are fewer organisms. In fact, apparent refractory MAC infections are commonly caused by reinfection with new strains rather than the original strain, which may explain the quite infrequent development of macrolide resistance (36). Therefore, a short *diagnostic* empiric trial of azithromycin monotherapy for 3 months for patients with non-cavitary minimally symptomatic disease to elicit a favorable radiographic response verifying a benign diagnosis is reasonable and safe and *may prevent the need for a major invasive procedure, including unnecessary surgical resection for an infection.*

### 3.3 Does every patient with known MAC need treatment?

Although the literature reports pulmonary MAC cure rates of 32–65% with 12-month macrolide-containing triple-drug regimens, there are high rates of drug intolerance (43%) (37) and recurrent disease, usually from reinfection with different strains and not treatment failures (30, 36, 38). The majority of the drug intolerance leading to discontinuation of therapy is from the rifampin adverse effects. In fact, there is good evidence that rifampin can be omitted so that a two-drug regimen of azithromycin and ethambutol works equally well for MAC treatment (as seen in Case 3) without the frequent and serious rifampin toxicity (39).

The macrolide favored almost all the time now is azithromycin, with a single-day dosage and rare side effects, unlike the poorly tolerated clarithromycin. Many regulatory agencies, such as the U.S. Food and Drug Administration, warn of the potential risk of ventricular arrhythmias with macrolide antibiotics due to potential elongation of the corrected QT interval (QTc) on the electrocardiogram. This risk is quite overstated, and numerous studies strongly refute this risk in older patients (>65 years), even those with chronic kidney disease, congestive heart failure, coronary artery disease, and concurrent use of a drug known to prolong the QT interval (40–42). A 2015 study by M. Trac et al. in a very large population-based retrospective propensity-matched cohort study in Ontario found no higher risk of ventricular arrhythmias in 616,359 patients receiving macrolides versus 705,132 patients receiving non-macrolide antibiotics (amoxicillin, cefuroxime, or levofloxacin) (40).

However, in immunocompetent patients with good nutritional status, no AIDS, minimal or no symptoms, and without fibro-cavitary or nodular bronchiectatic disease, the diagnosis of MAC does not require immediate initiation of treatment. As many as 40–60% of patients with MAC remain free of progression for years without treatment. Furthermore, 40–50% of patients with untreated MAC achieve spontaneous culture-negative conversion without antibiotic treatment. “Therefore, to avoid unnecessary treatments that might cause unwarranted medical expenses and adverse drug reactions, clinicians should consider the risk of disease progression and make timely decisions in the treatment initiation phase.” (38).

Given the complexity of correctly identifying these infections, understanding the varying clinical presentations of non-tuberculous mycobacterial lung diseases is becoming increasingly important. The infections presented in this report were largely asymptomatic or presented only with a slight productive cough but were initially suspected of being malignancies. In two cases, these infections were discovered incidentally during a screening procedure for a suspected lung carcinoma.

In many such cases, suspicion of malignant nodules can lead to otherwise unnecessary aggressive procedures, such as the upper lobectomy that was almost performed on the patient in Case 1. Fortunately, the results of the bronchoalveolar lavage culture prevented an unnecessary and potentially harmful procedure. Similarly, Case 2 avoided adverse treatment, even though the infection could have been mistaken for lung cancer based on the initial imaging. Additionally, considering that the patient in Case 3 had already received radiation therapy for a suspected lung malignancy, although it was likely MAC, it is fortunate that she underwent a core biopsy before receiving unnecessary additional radiation. In each of these cases, the patient was successfully treated medically, but the ability of MAC infections to mimic lung cancer on imaging necessitates awareness of both the prevalence and clinical manifestations of this disease.

### 3.4 Limitations

These three cases are not unique in our Center but rather illustrate the various presentations of MAC seen *commonly* in our Indeterminate Lung Nodule Clinic that are suspected of being lung cancer, particularly by the community providers. Although one patient was followed for 6 years without a recurrence, the other two cases were followed for less than 1 year each, so recurrent MAC or development of lung cancer is still possible. Fortunately, all three patients tolerated the drug therapy without significant side effects. Two patients were exceedingly pleased that they did not require surgery as initially recommended, and the other patient was delighted she did not need to undergo radiation therapy.

## 4 Conclusion

Many non-malignant etiologies are capable of mimicking lung cancer and can lead to invasive, expensive workups and surgery. Pulmonary nodules caused by non-tuberculous mycobacteria are one of the most common mimics of lung cancer. Recognizing the classic patterning of peripheral nodules in a tree-in-bud pattern along with bronchiectasis and mucous plugs on chest CT scans, combined with the rapid development in the size and number of nodules, can suggest an infectious etiology such as MAC pneumonia. If there is a high suspicion of MAC by a highly experienced pulmonary physician, then it is reasonable to proceed with an empiric 3-month course of azithromycin *without* an initial biopsy, followed by re-imaging. Due to its immunomodulating properties to reduce inflammation and its consistent mycobactericidal activity, a 3-month trial of empiric azithromycin may reduce nodule size and confirm a non-cancer etiology. This therapy also has a low risk of promoting macrolide resistance should further non-tuberculous mycobacterial therapy be needed. In the end, this practice has reduced unnecessary non-therapeutic surgery and invasive needle biopsy and provided relief from the anxiety of having a cancer diagnosis. Follow-up with regular chest CT scans for life can further alleviate fears of co-existing cancer and monitor possible progression of chronic pulmonary MAC infections that may warrant subsequent aggressive antibiotic treatment.

## Data Availability

The original contributions presented in the study are included in the article/supplementary material, further inquiries can be directed to the corresponding author.
